# Observation of yttrium oxide nanoparticles in cabbage (*Brassica oleracea*) through dual energy K-edge subtraction imaging

**DOI:** 10.1186/s12951-016-0175-z

**Published:** 2016-03-25

**Authors:** Yunyun Chen, Carlos Sanchez, Yuan Yue, Mauricio de Almeida, Jorge M. González, Dilworth Y. Parkinson, Hong Liang

**Affiliations:** Materials Science and Engineering, Texas A&M University, College Station, TX 77843-3123 USA; Mechanical Engineering, Texas A&M University, College Station, TX 77843-3123 USA; Department of Plant Science, California State University, Fresno, CA 93740 USA; Advanced Light Source, Lawrence Berkeley National Laboratory, Berkeley, CA 94720 USA

**Keywords:** Synchrotron X-ray micro-tomography, K-edge subtraction imaging, Yttria nanoparticles, Cabbage, Accumulation

## Abstract

**Background:**

The potential transfer of engineered nanoparticles (ENPs) from plants into the food chain has raised widespread concerns. In order to investigate the effects of ENPs on plants, young cabbage plants (*Brassica oleracea*) were exposed to a hydroponic system containing yttrium oxide (yttria) ENPs. The objective of this study was to reveal the impacts of NPs on plants by using K-edge subtraction imaging technique.

**Results:**

Using synchrotron dual-energy X-ray micro-tomography with K-edge subtraction technique, we studied the uptake, accumulation, distribution and concentration mapping of yttria ENPs in cabbage plants. It was found that yttria ENPs were uptaken by the cabbage roots but did not effectively transferred and mobilized through the cabbage stem and leaves. This could be due to the accumulation of yttria ENPs blocked at primary-lateral-root junction. Instead, non-yttria minerals were found in the xylem vessels of roots and stem.

**Conclusions:**

Synchrotron dual-energy X-ray micro-tomography is an effective method to observe yttria NPs inside the cabbage plants in both whole body and microscale level. Furthermore, the blockage of a plant’s roots by nanoparticles is likely the first and potentially fatal environmental effect of such type of nanoparticles.

## Background

Engineered nanoparticles (ENPs) have attracted great interests in commercial applications due to their unique physical and chemical properties [1]. Increased usage of ENPs has raised concerns in the probability of nanoparticles exposure to environment and entry to food chain [2]. The potential health and environmental impact of ENPs need to be understood [3, 4].

Plants are essential components of ecosystems and they not only provide organic molecules for energy but they can also filter air and water, removing certain contaminants [5]. Definitively, plants play a very important role in uptake and transport of ENPs in the environment [6]. Once ENPs are uptaken by plants and translocated to the food chains, they could accumulate in organisms and even cause toxicity and bio magnification [7, 8]. Nanoparticles are known to interact with plants and some of those interaction have been studied to understand their potential health and environmental impact, including quantum dots [9], zinc oxide [10], cerium oxide [11], iron oxide [12], carbon nanotubes [13], among others [14, 15]. The uptake of various ENPs by different plants was summarized in Table 1. Nanoparticles are known to stimulate morphological and physiological changes in several edible plants [16]. Hawthorne et al. noted that the mass of Zucchini’s male flowers were reduced by exposed to CeO_2_ NPs [11]. Quah et al. observed the browner roots and less healthy leaves of soybean treated by AgNPs, but less effects on wheat treated under same condition [15]. Qi et al. reported that the photosynthesis in tomato leaves could be improved by treated with TiO_2_ NPs at appropriate concentration [17].

**Table 1:** The uptake of different ENPs by plants

ENPs	Plants	Uptake	Ref.
NaYF_4_:Yb, Er	Pumpkin seedlings *(Cucurbita maxima)*	Root/stem/leaf	[8]
CdSe/ZnS QDs	A. Thaliana plant	Root	[9]
ZnO	Maize *(Zea mays L.)*	Root	[10]
CeO_2_	Zucchini *(Cucurbita pepo* *L.)*	Root/stem/leaf/flower	[11]
Fe_3_O_4_	Pumpkin (*Cucurbita* *maxima*)	Root/stem/leaf	[12]
C_70_	Rice *(Oryza sativa L.)*	Root/stem/leaf	[13]
AuNPs	Rice *(Oryza sativa)*	Root/shoot	[14]
	Radish *(Raphanus sativus)*	Root	
	Pumpkin (*Cucurbita* *maxima*)	Root	
	Ryegrass *(Lolium perenne* *L.)*	Root/shoot	
AgNPs	Soybean *(Glycine max)*	Root/shoot	[15]
	Wheat *(Triticum aestivum)*	Root/shoot	

Yttrium oxide (Y_2_O_3_, yttria) ENPs have been broadly used in optics, electrics and biological applications due to their favorable thermal stability and mechanical and chemical durability [18–20]. One of the most common commercial applications is employed as phosphors imparting red color in TV picture tubes. The environmental effects of yttria ENPs have not been reported. Even though the effects of certain NPs have been studied on several plants [14], the uptake, translocation and bioaccumulation of yttria NPs in edible cabbage (Brassicaceae, *Brassica oleracea*) have not been addressed until this study. This plant species was chosen and tested as part of a closed hydroponic system designed to study nanoparticles movement and distribution in a substrate-plant-pest system as a model of a simple and controlled environment. The final test “substrate” used was plain distilled water (to avoid NPs to attach or react with other substrate elements), in which the tested NPs were mixed.

In order to observe the translocation and distribution of ENPs in plants, transmission electron microscopy (TEM) has been one of the most commonly used techniques to identify the localization at cellular scale in two-dimensions (2D), because it can be used to observe all kinds of ENPs [21, 22]. On the other hand, ENPs with special properties, such as upconversion NPs and quantum dots with a particular band gap can be studied with a confocal microscope with alternative excitation wavelengths to trace the ENPs [8, 23]. Several synchrotron radiation imaging techniques exploiting high energy X-ray have become widely used in plant science, which can measure both spatial and chemical information simultaneously, like micro X-ray fluorescence and computed tomography [24–26].

In this research, we use synchrotron X-ray micro-tomography (µ-XCT) with K-edge subtraction (KES) to investigate the interaction of yttria NPs with edible cabbage. By using the KES technique, the µ-XCT can not only detect the chemical and spatial information in 3D, but also analyze the concentration of target NPs. The uptake, accumulation, and distribution mapping of yttria NPs in both micro scale and relatively full view of cabbage roots and stem were investigated. We found that yttria NPs were absorbed and accumulated in the root but not readily transferred to the cabbage stem. Compared with yttria NPs, other minerals were observed along the xylem in both cabbage roots and stem. To the best of our knowledge, few reports have studied the impact of yttria NPs on cabbage plants. In addition, by using µ-XCT with KES technique, the distribution and concentration mapping of nanoparticles in full view of plant root have not been previously reported.

## Results and discussion

### Physical properties of yttria nanoparticles

**Fig. 1:**
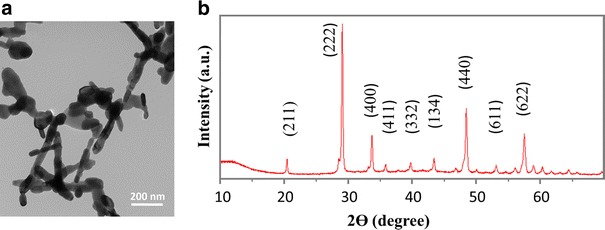
**a** TEM image and (**b**) XRD pattern of as-synthesized yttria NPs

The yttria NPs were characterized by using TEM and XRD (Fig. 1). The mean diameter of nanotubes is 31.3 ± 8.6 nm, and the mean length is 206.3 ± 77.3 nm. The average size of irregular nanoparticles is 64.9 ± 16.9 nm (Fig. 1a). The XRD pattern of as-synthesized NPs was finely indexed to a cubic phase of yttria (JCPDS card no. 83-0927), shown in Fig. 1b. The as-calcined yttira NPs did not have further surface modification, therefore, the NPs were not water-soluble.

### Identifying nanoparticles in cabbage

**Fig. 2:**
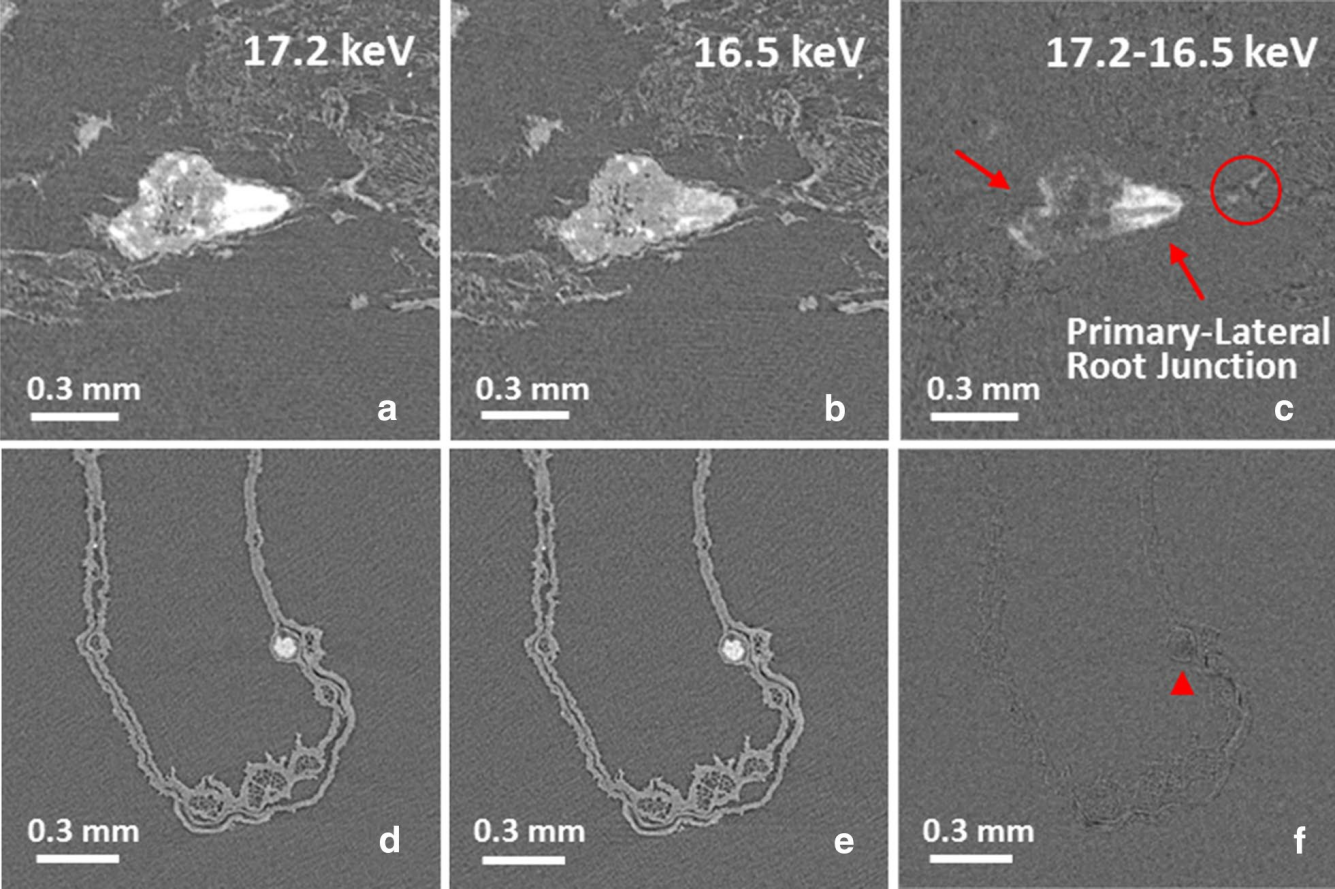
Transverse reconstructed slices at the junction between primary root and lateral root scanned at (**a**) 17.2 keV, (**b**) 16.5 keV, and (**c**) subtracted slice. (**d–e**) The slices for leave section

The µ-XCT was carried out at Beamline 8.3.2 at the advanced light source, Lawrence Berkley National Laboratory. From scanning energies of 16.5 to 17.2 keV, below and above yttrium K-edge, the X-ray attenuation coefficient sharply increases by a factor of 5. Other elements decrease slightly in their attenuation coefficients over this energy range. The localization of yttria NPs can be identified by the subtraction between two reconstructed image datasets (17.2–16.5 keV), shown in Fig. 2. The slices collected above and below the K-edge were set with same brightness and contrast settings to fairly compare with each other. The grayscale values of reconstructed slices represent the absorption coefficient; therefore, the bright regions in subtracted slice denote the localization of yttria NPs (Fig. 2c arrowed). Other elements appear dark in subtracted slice marked with a red “▲” (Fig. 2f). These are inorganic elements which support the growth of cabbage. Some biological structures suffered radiation damage during scanning, resulting in a small amount of shrinkage. The bright regions circled in Fig. 2c were caused by such shrinkage, resulting in a registration mismatch between the images above and below the edge. To identify and map the distribution of yttria NPs, an image segmentation protocol was employed that could highlight regions with yttria without finding these regions corresponding to sample shrinkage. The detailed segmentation process is given in the “Method” section.

### Three-dimensional distribution and quantification of nanoparticles in cabbage

**Fig. 3:**
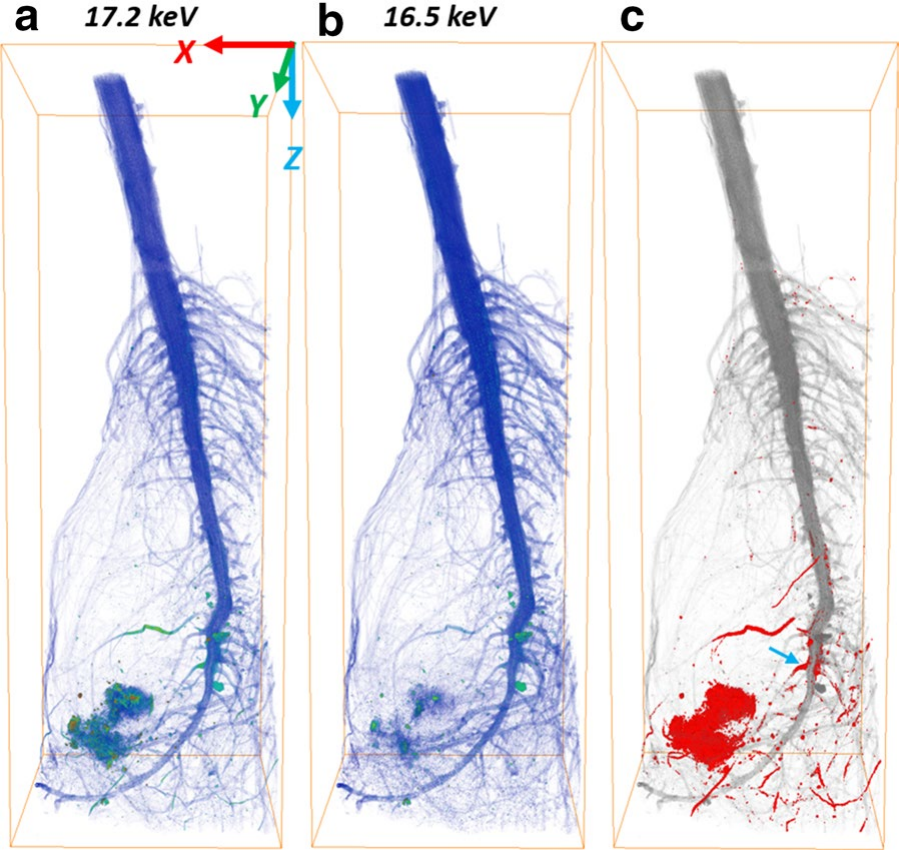
The 3D visualization for a wide view of root built by (**a**) 17.2 keV transverse reconstructed slice datasets and (**b**) 16.5 keV datasets. (**c**) Distribution of yttria NPs (*red*) in root. The *grey* visualization was built by 17.2 keV; the *red* one was built by the subtracted datasets. The bounding box size is 6.77 × 5.10 × 19.40 mm

By using K-edge subtracted image technique with Monochromatic X-ray tomography, the translocation and distribution of NPs in the cabbage root is clear (Fig. 3). Figure 3a and b were constructed by 17.2 keV and 16.5 keV reconstructed slice datasets, respectively. Their color maps were based on the transverse slice pixel values/absorption coefficients over the range from 0.2 to 17.8 cm^−1^. An obvious difference between 17.2 and 16.5 keV visualization in absorption coefficient of yttria NPs was observed. The distribution of yttria NPs in root was segmented and colored in red (Fig. 3c). A large amount of NPs were found aggregated at left bottom of the root. Since yttria NPs were not water-soluble, the water that contained them was kept in constant movement with an air pump working 24/7. However, it seems that the dense roots formed a web-like structure that made the suspended NPs to accumulate and aggregate among the roots. Uptake of NPs by the root has been observed at primary and lateral root junction as well according to the transverse slice. Figure 2a is one transverse slice localized at the arrow in Fig. 3c (blue arrow) showing the junction between primary root and lateral root. We found that the yttria NPs were absorbed by the lateral roots, and particulates began to accumulate along the outer epidermis of primary roots with limited entrance into the vascular tissue (xylem and phloem) of the primary root. It might happen that endodermal cell walls were blocking the entrance of aggregated yttria NPs into vascular tissue [10]. This is shown in the upper section of the 3D visualization (Fig. 3c) where no yttria NPs were observed above the root system.

**Fig. 4:**
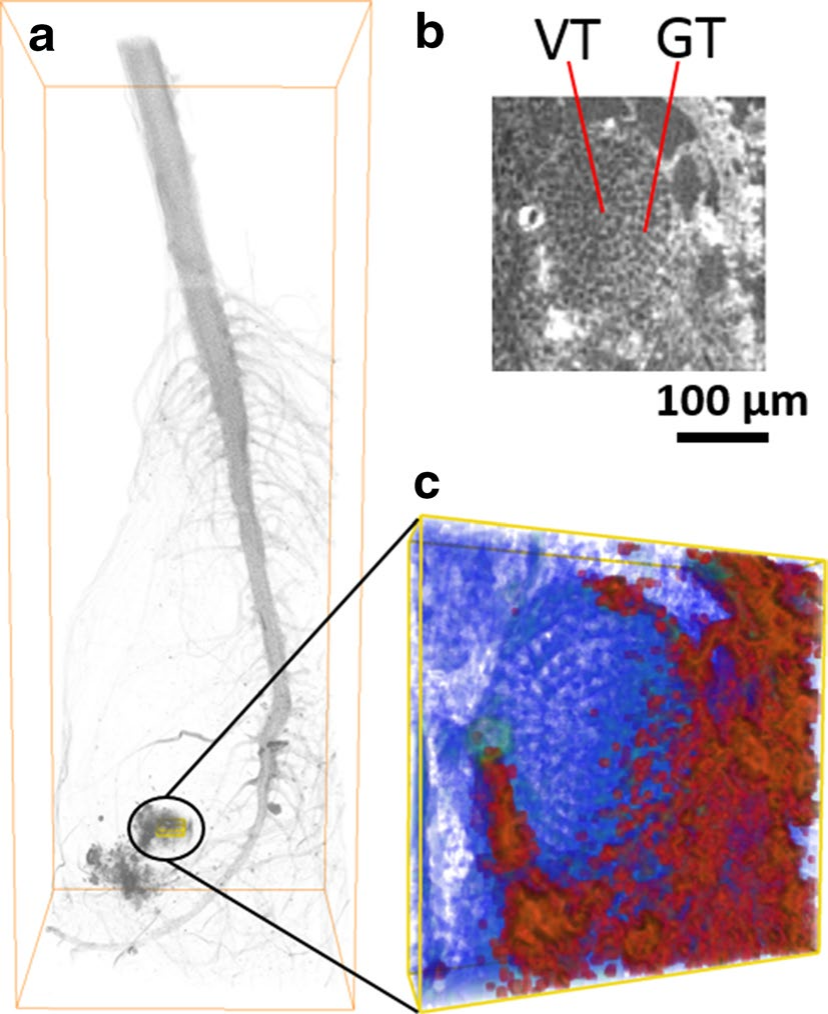
The micro-scale of root segmentation localized at yttria NPs aggregated regions. **a** The selected *yellow* region in 17.2 keV visualization. **b** The top transverse slice of *yellow* frame region. **c** Magnified view of *yellow* frame. The vascular tissue (VT) and ground tissue (GT) are shown in (**b**). The *red* regions in (**c**) show the distribution of yttria NPs. The *yellow* frame size is 0.58 x 0.58 x 0.13 mm

Besides the full view of the translocation in the cabbage root system, the distribution of yttria NPs at the micro-scale within a lateral root was detected and investigated (Fig. 4). Figure 4a shows the localization of the micro-scale lateral root visualization. The 3D visualization of micro-scale was built by the segmented transverse reconstructed slices, and the red regions were localized yttria NPs (Fig. 4b, c). It is clear that roots are able to uptake the yttria NPs in ground tissue (GT), which appear to accumulate in the root with limited entrance of yttria NPs into vascular tissue (VT) being transported through the xylem. Xylem vessels are small with diameters usually smaller than 1 μm in vegetables like cabbage plants to over 100 μm in vessels found in trunks of large trees [27]. Vessels allow nutrients contained in water to be distributed throughout the plant. For NPs, however, if they aggregate, the blockage is expected, that is what we have observed in this study. Long term studies might show that yttria NPs might provide more negative than positive effects on plant growth and development as found with other NPs (i.e., AuNPs, AgNPs) [16].

**Fig. 5:**
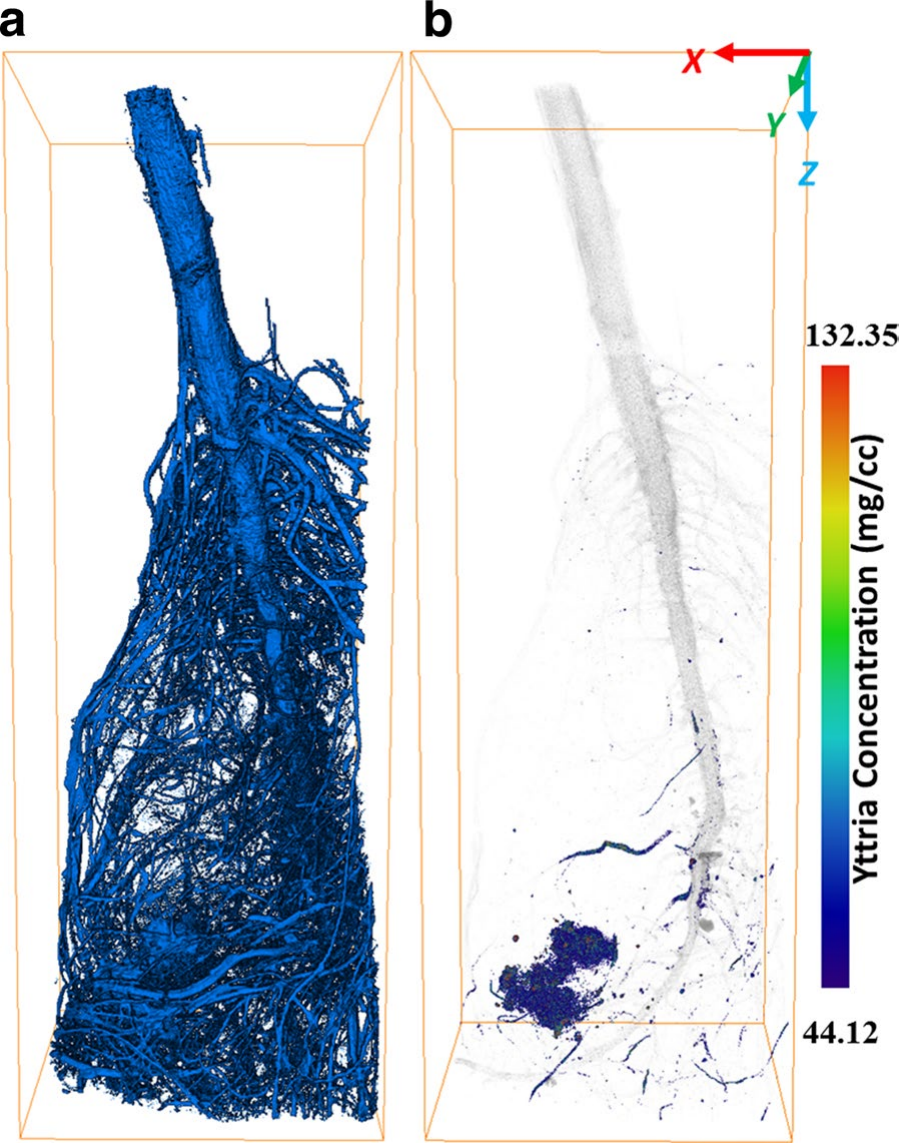
(**a**) The full volume visualization of plant root. (**b**) Concentration map of yttria NPs in root (on the voxel level)

Using K-edge subtraction image technique with dual-energy X-ray scanning, the concentration of target NPs can be calculated. This method has been discussed elsewhere [28–30]. As attenuation coefficients of other elements just have a slight decrease, the concentration (C_NPs_) can be formulated in a simplified equation

$$C_{{NPs}} = \frac{{\Delta \mu \left( {x,y} \right)}}{{\frac{\mu }{\rho }\left( {17.2} \right) - ~\frac{\mu }{\rho }(16.5)}},$$

where Δµ is the difference in absorption coefficient obtained by subtraction between two energies, µ/ρ is the mass absorption coefficients. The value for Δµ is obtained from the voxel value of subtracted datasets, and the mass absorption coefficient is from Argonne National Labratory (Compute X-ray Absorption). The volume rendering enable the 3D visualization for the concentration map of yttria, shown in Fig. 5b. By using this formula, the calculated concentration is based on the voxel level. The minimum concentration was 44.12 mg/cc and the maximum was 551.47 mg/cc (to display the mapping colorful, the maximum set as 132.35 mg/cc). The grey visualization (setting 30 % transparent) of root shows the distribution and localization of yttria NPs. Using Avizo software with image segmentation and label-analysis, the total voxel volume of root is measured as 5.41604e + 07 voxels. Figure 5a shows the full volume of the root section. As shown the concentration of nanoparticles at root was estimated in the range from 0.82 to 10.18 µg/L.

**Fig. 6:**
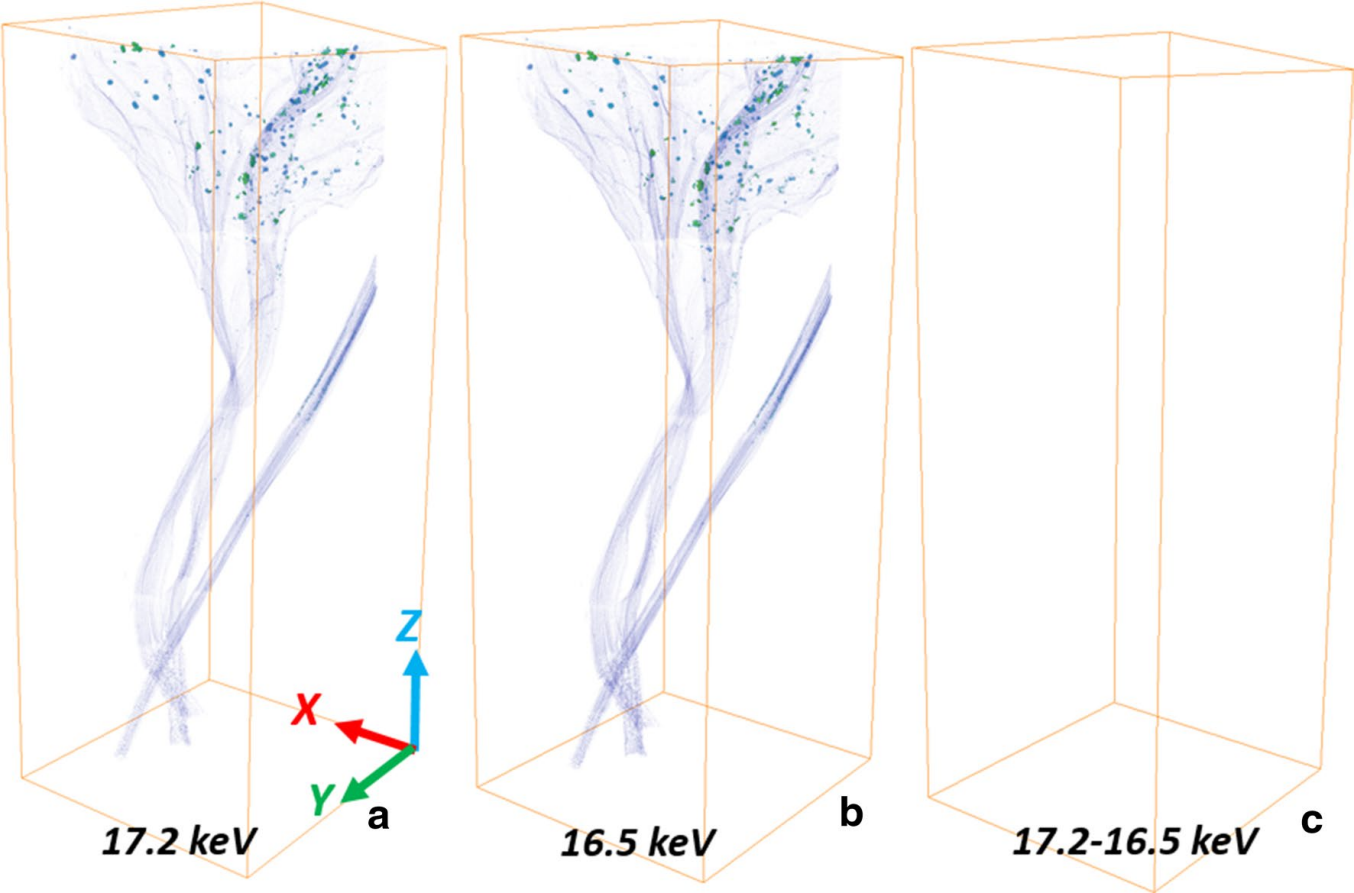
The 3D visualization of shoot section for cabbage built by (**a**) 17.2 keV transverse reconstructed slice datasets and (**b**) 16.5 keV datasets. (**c**) Distribution of yttria NPs in shoot. The bounding box size is 7.65 × 7.29 × 19.40 mm

For the cabbage shoot, no yttria NPs were observed (Fig. 6), which means that no yttria NPs transported from roots to shoots. As we found no yttria NPs entering vascular tissues of primary root, the yttria NPs accumulated making it difficult to be transported by xylem from the root to the rest of the plant. Despite no clear evidence of yttria translocation, other elements were observed in the shoots. In general, the higher the atomic number (Z), the higher the absorption coefficient for a given X-ray energy (Fig. 2d–f). It is clearly to see some high-Z (compared with carbon) elements distributed in both roots and shoots. Crops require many mineral elements for their growth, such as calcium, magnesium, zinc, copper and iron [31, 32]. These high-Z elements could be the mineral elements absorbed by cabbage before the cabbage root exposed in the hydroponic system containing yttria NPs.

What are the possible uptake mechanisms based on the observation? As shown above, we observed that some yttria NPs were uptaken by the roots of cabbage plant (Figs. 2a–c and 4). The cell wall is considered as a tight and significant sieve which blocks the migration of NPs [33]. The typical pore sizes of a cell wall are in the range of 2–20 nm [2]. In our case, the yttria NPs sizes are larger than the pore sizes, therefore, the passage for NPs through the pores of cell walls should be difficult. On the contrary, the larger NPs were found to be taken up by roots or shoots that are in correlation with previous reports [12, 34, 35]. It is not that clear which route NPs can penetrate the cell wall for all these cases. Shen et al. reported that an endocytosis-like structure was observed in *Arabidopsis thaliana* leaf cells [36]. Therefore, the yttria NPs could penetrate the cell wall and be taken up by the roots. In addition, the dissolution rates of rare earth oxides are too low to be relevant [37]. Even though some yttria NPs penetrated into the ground tissues, the yttrium ions were not established. This could be the reason the yttria NPs only observed in ground tissue and blocked at primary-lateral-root junction.

### Limitations in KES

Although a KES method can identify the localization of target NPs, if the concentration of root-to-shoot-transported yttria NPs was too low, the target NPs could not be detected. Furthermore, the KES method is based on the difference in attenuation coefficient of yttrium element over K-edge. This method is able to identify the yttrium-based NPs but it cannot distinguish the biotransformation of yttria.

## Conclusions

Synchrotron µ-XCT with KES image technique is a valid method to study the uptake, accumulation and spatial distribution mapping of yttria NPs in cabbage roots. Using the KES technique, the concentration mapping of yttria NPs was calculated and shown in 3D visualization. The yttria ENPs were uptaken by root but not found in the cabbage shoot. Instead, other non-yttria minerals were found in both cabbage root and shoot. The blockage of yttria NPs was mainly due to their accumulation at primary-lateral-root junctions.

## Methods

### Synthesis and characterization of nanoparticles

The Yttria nanoparticles were synthesized by using a hydrothermal method [38]. All chemicals were Sigma-Aldrich (USA). The 5.94 mmol Y_2_O_3_ and 0.02 mmol Al_2_O_3_ powders were dissolved in 250 mL HNO_3_ solution (2.8 wt %) to attain a transparent solution at 60 °C, followed by adding 0.06 mmol Er(NO_3_)_3_·5H_2_O and 0.06 mmol Yb(NO_3_)_3_·5H_2_O. By adding the 3 M KOH solution into the transparent solution, the solution pH value was adjusted to 10.5. When pH value was over 7, the white floccules were appeared. The obtained turbid solution was 900 mL. After stirred 10 min, the turbid solution was transferred to a 2 L general purpose pressure vessel and heated at 200 °C for 12 h without stirring. After cooling down to room temperature, the precipitate was attained by centrifuging at 5000 rpm for 15 min, continued by washing with DI water. The final Yttria-based NPs powders were acquired by drying the precipitate at 60 °C and heating the dried precipitate at 1000 °C for 3 h in the air.

A transmission electron microscopy (TEM, JEOL 1200 EX) was used to image the as-synthesized yttria NPs, using an accelerating voltage of 100 keV. The crystal structure of yttria NPs was measured by a Bruker-AXS D8 Advanced Bragg–Brentano X-ray powder diffractometer (XRD) operated at 40 mA and 40 kV with with Cu Kα radiation (λ = 1.5418 Å).

### Cabbage culture and exposure to nanoparticles

**Fig. 7:**
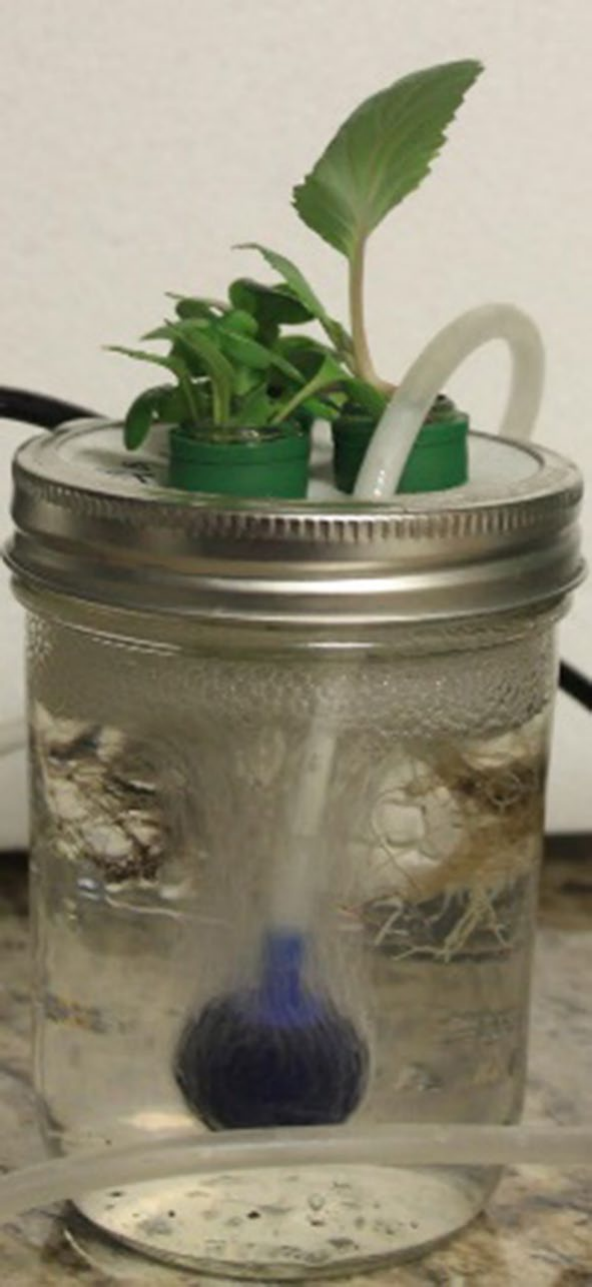
Hydroponic system for cabbage exposure to yttria NPs

Cabbage plants were reared in a hydroponic system as shown in Fig. 7. Seeds of cabbage were placed in 38 mm compressed (100 % peat) plugs and placed in a hydroponic mix containing water to which a 2-1-2 (NPK) solution (118 mL per 20 gallons of water) was added every week. Once plants had four true leaves, they were extracted from the main culture system, cleaned and placed into two groups. One group was placed in a glass jar (1 pint) containing distilled water and yttria NPs (10 plants per jar), the other was placed in only distilled water (10 plants per jar; as control). The 0.120 g NPs were added to distilled water in a small Nalgene container, mixed with a mini vortexer, and then added to the distilled water up to 0.38 L in the final testing glass jar. All jars had an air pump in them which were running 24/7. The distilled water inside the glass jars containing NPs were kept in movement with the air pump working 24/7. NPs did not form conglomerates in the hydroponic testing system. The “substrate” used was plain distilled water (to avoid NPs to attach or react with other substrate elements), in which the tested NPs were mixed. Even though both groups showed clear sign of stress after 10 days, they were maintained in this system for a total of 22 days. About 30 % of the plants tested (with and without NPs) were wilted, the plants that were in better shape were collected, cleaned thoroughly with distilled water, dried and fixed with Kahle’s, a fixing agent that provides sharp and clear preservation of nuclear structure of plant or animal tissues. Once received for imaging, plants were extracted from the container and let dried before placing them in the Synchrotron X-ray micro-tomography equipment.

### Synchrotron X-ray micro-tomography

**Fig. 8:**
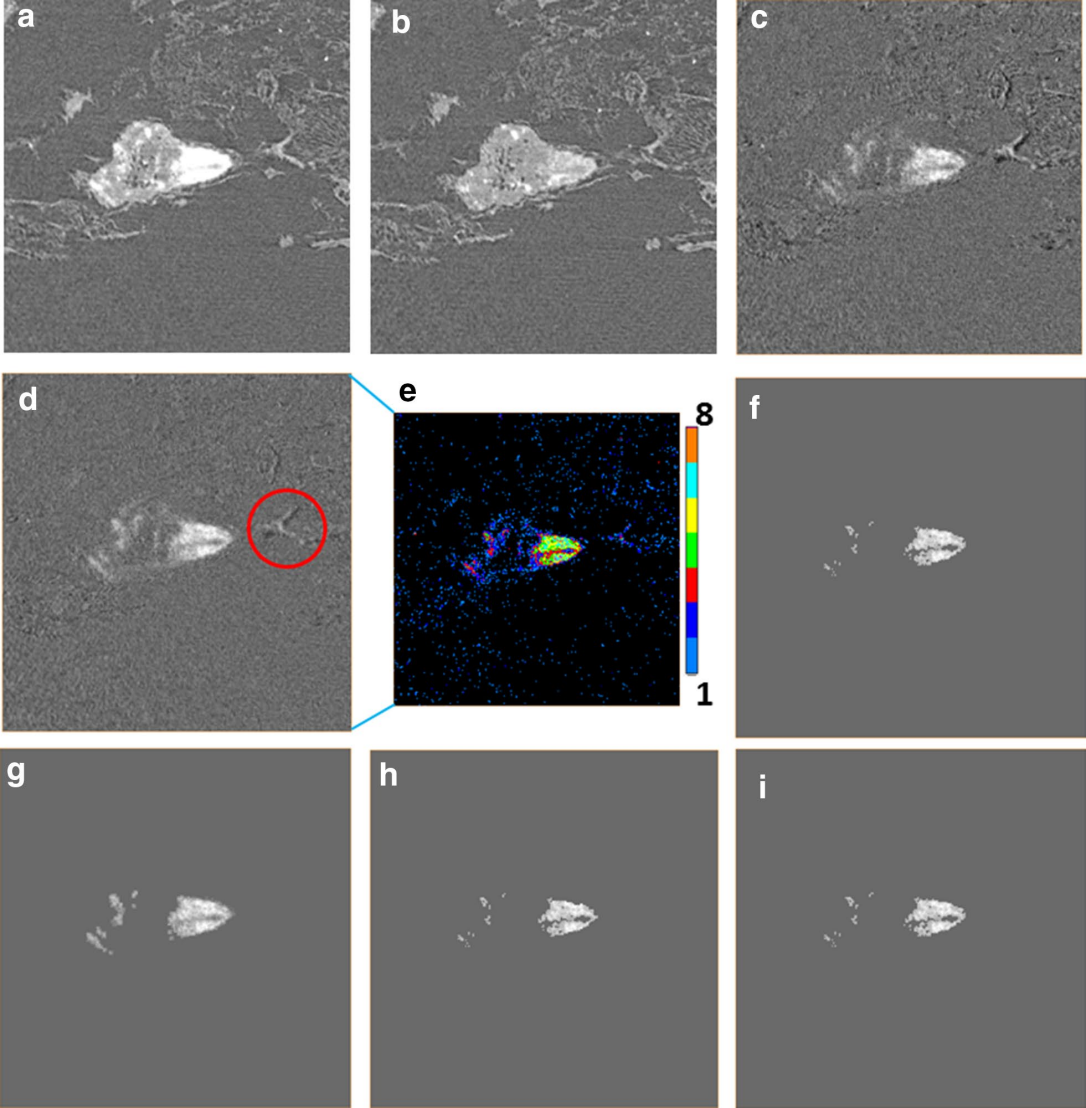
The process of image segmentation. **a** Reconstructed image obtained 17.2 keV. **b** Reconstructed image at 16.5 keV. **c** The subtracted slice from (**a**) and (**b**). **d** Subtracted slice with an image registion fucntion. **e** Colors lable the intensity of pixel value of (**d**). **f**–**i** Image segmentation based on the threshold of (**a**, **b**). Detailed data of images (**f**–**i**) is listed in Table 2

Synchrotron X-ray computed micro-tomography was conducted at Advanced Light Source beamline 8.3.2 facility, Lawrence Berkeley National Laboratory. Monochromatic X-ray at 16.5 and 17.2 keV were employed with calibration for transmission of yttria NPs at approximately 67 and 15 %, respectively. Radiographs were acquired by using a LuAG scintillator, 2× optical lens, and PCO_Edge scientific CMOS camera, yielding a pixel size of 0.00319 mm. The cabbage specimen was irradiated with 200 ms exposure time per frame and rotated over 180° with 512 projections. The datasets were reconstructed by (Fig. 8) using a Fourier method implemented in the commercial Octopus package and further processed using ImageJ. To investigate the translocation of NPs in cabbage, the three-dimensional (3D) visualization was built with Avizo software (FEI).

### Image segmentation

**Fig. 9:**
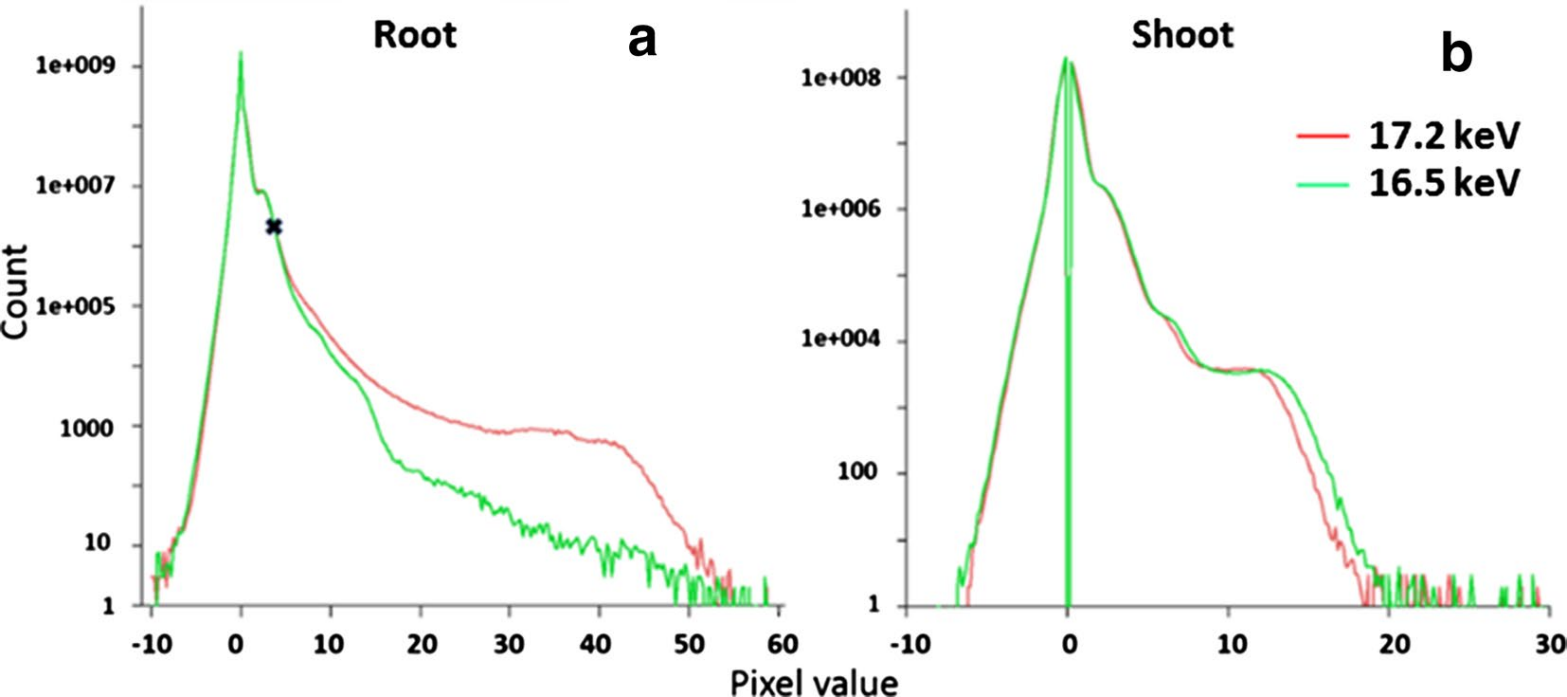
Histograms of image pixel value (absorption coefficient) for (**a**) root and (**b**) shoot datasets

The image segmentation was carried out by Avizo software to identify and display the distribution of NPs in 3D visualization. Figure 8 takes a root section image as an example to show the procedure and changes of segmentation. Figure 8a and b were the transverse slices scanning at 17.2 and 16.5 keV. Though the dual-energy slice datasets were scanned at the same anatomic location, the slight shrinkage and shift of the biological structure could take place during the hard X-ray radiation. Image registration was firstly employed to compensate for such shift and obtain the better quality of subtracted slices. Figure 8c and d are the subtracted slices obtained without and with image registration, respectively. The 17.2 keV reconstructed datasets were thresholded with the pixel value 3.8 corresponding to the Fig. 9 at marker “X”, with count of 17.2 keV datasets more than that of 16.5 keV (Threshold A). The subtracted datasets were thresholded with three according to the Fig. 8e, as the pixel values less than 3 (light and deep blue labels) could be caused by the organic plant body or the noise (Threshold C). All pixel values above the threshold were labeled as 1, with candidate NPs; whereas non-labeled areas were set as 0. The shift due to the sample motion or shrink during scanning can be identified by the regions of increased darkness adjacent to regions of increased brightness (Fig. 8d circled). The darkness regions (pixel value less than 0) will be selected and dilated in 3D with 26 adjacent voxels. The dilation regions were labeled as one (Threshold D). Figure 8f was derived by arithmetic with Threshold A, C and D as Table 2 shows. Figure 8g is the dilation of Fig. 8f with 26 adjacent voxels in 3D. Figure 8h was obtained by removing the pixel value over three in Fig. 8g (Table 2). The final segment was derived via subtracting the non-yttria regions which were generated by the shifting of high-Z elements (Fig. 8i). The arithmetic was with Threshold B, E and Fig. 8h (Table 2).

**Table 2:** The characterizations for the sub-Fig. 8

	Characterization
Figure 8a	17.2 keV; Threshold A: pixel value no less than 3.8; Threshold B: no less than 12
Figure 8b	16.5 keV; Threshold C: no less than 3; Threshold D: dilation for less than 0; Threshold E: between 3 and 4
Figure 8c	17.2–16.5 keV without registration
Figure 8d	17.2–16.5 keV with image registration
Figure 8e	Labeled color map due to pixel value
Figure 8f	Threshold A × C−D
Figure 8g	Dilation of Fig. 8f
Figure 8h	Figure 8g × Threshold C
Figure 8i	Figure 8h−(Threshold B × C)

## Abbreviations

ENPs: engineered nanoparticles
TEM: transmission electron microscopy
Y_2_O_3_/Yttria: yttrium oxide
2D: two-dimensional
3D: three-dimensional
µ-XCT: X-ray micro-tomography
KES: K-edge subtraction
XRD: X-ray diffractometer
GT: ground tissue
VT: vascular tissue
