# Erratum to: Observation of yttrium oxide nanoparticles in cabbage (*Brassica oleracea*) through dual energy K-edge subtraction imaging

**DOI:** 10.1186/s12951-016-0186-9

**Published:** 2016-04-20

**Authors:** Yunyun Chen, Carlos Sanchez, Yuan Yue, Mauricio de Almeida, Jorge M. González, Dilworth Y. Parkinson, Hong Liang

**Affiliations:** Materials Science and Engineering, Texas A&M University, College Station, TX 77843-3123 USA; Mechanical Engineering, Texas A&M University, College Station, TX 77843-3123 USA; Department of Plant Science, California State University, Fresno, CA 93740 USA; Advanced Light Source, Lawrence Berkeley National Laboratory, Berkeley, CA 94720 USA

## Erratum to: J Nanobiotechnol (2016) 14:23 DOI 10.1186/s12951-016-0175-z

After publication of this article [1], we received support to pay the article processing charge. Part of the open access publishing fee for this manuscript has been provided by the Texas A&M University Online Access to Knowledge Fund (OAKFund), supported by the University Libraries and the Office of the Vice President for Research.
